# Ileosigmoid Knotting: Analysis of Patients Clinical Profiles and Determinants of Outcomes

**DOI:** 10.1155/2020/3826138

**Published:** 2020-07-23

**Authors:** Kirubel Abebe, Kalid Sherefa, Henok Teshome, Engida Abebe

**Affiliations:** Department of Surgery, St. Paul's Hospital Millennium Medical College, Addis Ababa, Ethiopia

## Abstract

**Introduction:**

Ileosigmoid knotting (ISK) is an uncommon form of bowel obstruction due to wrapping of the ileum or sigmoid colon around the base of the other. It is associated with poor prognosis. Data on ISK are scarce in our country. The aim of this study was to assess clinical profiles, management, and outcome of patients operated for ISK. *Methodology*. A retrospective analysis of all patients operated for ISK at St. Paul's hospital millennium medical college (SPHMMC) from February 2014 to January 2020 was performed.

**Results:**

A total of 28 patients (M: *F* = 3 : 1) were studied. The mean age was 41.7 years (SD ± 19.5) and ranged from 18 to 80 years. The mean duration of illness was 1.6 days (SD ± 1.1). Abdominal pain and vomiting were seen in all patients followed by abdominal distention (24, 85.7%) and failure to pass feces or flatus (23, 82.1%). Preoperative diagnosis was correct in 6 (21.4%) patients. Almost all patients (26, 92.8%) had gangrenous bowel. The commonest procedure performed was resection of the gangrenous segments with primary ileoileal anastomosis and sigmoid end colostomy (16, 57.1%). Complications were seen in 11 (39.3%) patients and the commonest being surgical site infection (SSI) (7, 25%). Death occurred in 6 (21.4%) patients, and it was significantly (*p*=0.020) associated with intraoperative shock (systolic blood pressure (SBP) < 90 mmHg).

**Conclusion:**

ISK lacks specific clinical features and imposes a significant rate of bowel strangulation, which deserves high index of suspicion and urgent laparotomy. The choice of surgical procedure should be determined by intraoperative bowel status and patients' general condition.

## 1. Introduction

Ileosigmoid knotting (ISK) is an uncommon etiology of intestinal obstruction due to wrapping of the ileum or sigmoid colon around the base of the other resulting in double closed loop bowel obstruction [1–4]. The incidence is not well-known, but it is commonly seen in parts of the world where there is a high incidence of sigmoid volvulus (SV). It comprises around 18–50% and 5–8% of SV patients in Eastern and Western countries, respectively [4, 5]. It has a male predominance with a peak incidence at 3^rd^–5^th^ decades [2–9].

A long small bowel mesentery and freely mobile small bowel and a redundant sigmoid colon on a narrow mesenteric base are the major anatomic prerequisites for the occurrence of ISK. Other conditions such as consumption of a high bulk diet in the presence of an empty small bowel, late pregnancy, postoperative adhesions, malrotations, internal herniation, and Meckel's diverticulum can also predispose to ISK [1, 2, 4, 9, 10].

The diagnosis of ISK usually made intraoperatively due to its nonspecific presentation, clinical feature, laboratory, and imaging [1–4, 8–10]. Abdominal pain, failure to pass feces and/or flatus, abdominal distention, are vomiting are the major complaints. Signs of strangulation occur early due to the double closed loop nature of the obstruction [1, 2, 5, 9–15]. The common X-ray findings include distended large and small bowel loops with multiple fluid air level [1, 2, 4, 7–9]. Features on abdominal computed tomography (CT), ‘the whirl sign' created by the twisted intestine and sigmoid mesocolon, increase the diagnostic accuracy in more than 90% of the cases [1, 2, 7, 8, 14, 16]. In general, a clinical picture of small bowel obstruction, radiographic evidence of large bowel obstruction, and inability to insert an endoscope has been proposed as a useful diagnostic triad of ISK [2, 15].

Emergency laparotomy after adequate resuscitation is the standard of care for patients with ISK [1, 2, 4, 9]. The type of procedure depends on the bowel viability and patients' condition. It can range from detorsion alone for nongangrenous cases to resection of significant portion of the bowel for gangrenous bowel. Unfortunately, most of ISK cases had gangrenous bowel. Even though a variety of procedures are performed to manage strangulation in ISK, the preferred is resection of gangrenous segments with primary anastomosis of the ilium and sigmoid end colostomy. In addition, stomas (colostomy or ileostomy) after resection can be performed as a salvage procedure in patients with poor general condition or with bowel problem such as edema and size discrepancy between proximal and distal bowel diameters [1–3, 6, 8, 9, 14, 17–19].

ISK carries high morbidity and mortality due to the aggressive nature of the disease. Factors such as advanced age, late presentations and diagnosis, comorbidities, and pregnancy are associated with increased mortality [1–3, 10, 11, 13–15].

Because ISK is uncommon, there are no strong studies performed in Ethiopia to tell the real burden and nature of the disease. So far, to the best of the authors knowledge, only two institution-based studies were performed in Ethiopia with small sample size, and the recent study being fourteen years old [11, 12]. Conducting this study to assess the clinical profile, type of surgery and outcome determinants of patients operated for ISK will help to provide a recent data and that can be used as a guide to develop management protocol and for further studies.

## 2. Materials & Methods

A retrospective medical record review of all patients operated for ISK between February 2014 and January 2020 was performed at SPHMMC, Addis Ababa, Ethiopia, in May 2020. SPHMMC is a teaching referral hospital serving a catchment area of more than 6 million people. General surgeons and senior surgical residents (under strict supervision) were involved in the care of all patients in this study. The operation theater register was used to identify patients' medical records, which were the main source of data. A total of 34 patients operated for ISK were found, but only 28 records were identified due to incomplete charts or lost medical records. A data collection format which was tested on five patients was used to collect data on sociodemographic characteristics, clinical presentation, preoperative investigations, intraoperative findings, type of surgical procedure performed, and patients' treatment outcomes. Data were checked for completeness, accuracy, and consistency, and then coded, entered, and analyzed with SPSS version 23. Results were shown using tables and central tendency statistics. During the statistical test, associations were considered significant when *p* value was <0.05. A written ethical clearance letter was obtained from SPHMMC Institutional Review Board.

## 3. Results

A total of 34 patients were operated for ISK during the study period. Of these, medical records of 28 patients were reviewed and analyzed. Males were commonly affected with a male to female ratio of 3 : 1. The peak age for ISK was between 20 and 29 years. The mean age of the patients was 41.7 years (SD ± 19.5) and ranged from 18 to 80 years (Table 1). Only one of the patients had comorbidity and hypertension.

The average duration of illness was 1.6 days (SD ± 1.1), and the majority (25, 89.3%) presented within 48 hours. Three-fourths (15, 75%) of patients who reside in rural area presented later than 24 hours, while 7 (87.5%) of patients from urban settings presented within 24 hours. Abdominal pain and vomiting were the presenting symptoms in all patients followed by abdominal distention (24, 85.7%) and failure to pass feces or flatus (23, 82.1%). Five (17.9%) of the patients were in shock at presentation. The leading abdominal findings were tenderness (26, 92.8%), rebound tenderness with guarding (19, 67.8%), and hypertympanic abdomen (24, 85.7%). On digital rectal examination, stool was found in more than half (15, 53.6%) of the patients Table 2.

Of the patients, 13 (46.4%) had leukocytosis. Erect plain abdominal X-ray was obtained in 17 (60.7%) of the cases, and the remaining had no documentation if X-ray was requested or not. Among these, 7 (41.2%) patients had small bowel distension with multiple air fluid level, 5 (29.4%) patients had large bowel distension with multiple air fluid level, 3 (17.6%) patients had only large bowel distension, and only 2 (11.8%) patients had no clear features of obstruction.

Accurate diagnosis of ISK was made preoperatively only in 6 (21.4%) patients. The three top misdiagnoses were small bowel obstruction (14, 50%), sigmoid volvulus (4, 14.2%), and perforated viscus (2, 7.2%).  Table 3.

After adequate resuscitation with crystalloids and initiation of antibiotics, all patients underwent urgent laparotomy through vertical midline incision. Majority (24, 85.7%) had hemorrhagic peritoneal fluid. The active component was the ileum in 26 (92.8%) patients. Almost all patients (26, 92.8%) had gangrenous bowel at laparotomy. Of these, 17 (65.4%) involve both the ileum and sigmoid. The rate of gangrenous change was almost similar in those presented within 24 hours (11/12, 91.7%) and later (15/16, 93.7%). The commonest (16, 57.1%) procedure performed was resection of gangrenous segments with primary ileoileal anastomosis and sigmoid end colostomy.  Table 4.

Potential contributing factors identified intraoperatively include redundant sigmoid colon on a narrow mesenteric base (26, 92.8%), third trimester pregnancy (1, 3.6%), and previous surgery (1, 3.6%).

Complication was seen in 11 (39.3%) patients. The commonest postoperative complication was surgical site infection (SSI) (7, 25%). Four patients had deep SSI which required relaparotomy (Table 5). Acute renal failure (ARF) was noted in 2 (7.2%) patients who had shock either at presentation or intraoperatively. Grossly, the risk of complication was similar between patients who had double (7/17, 41.2%) and single (4/9, 44.4%) segment strangulation. But anastomotic leak was entirely seen in patients with double segment strangulation (3/17, 17.6%). New onset intraoperative shock was documented in 4 (14.3%) patients. Even if it is statistically insignificant, patients who were in shock intraoperatively had higher rate of anastomotic leak than patients who had no shock (1/4, 25% vs. 2/24, 8.3%, *p*=0.343).

The average duration of hospitalization was 10.7 days (SD ± 8.1) and ranged from 2 to 40 days. Death occurred in 6 patients making the mortality rate 21.4%. Multiorgan failure due to sepsis was the cause in all cases. All the mortalities occurred in patients with gangrenous bowel, either double segment (4/17, 23.5%) or single segment (2/9, 22.2%) Table 6.

Age, sex, residence, duration of presentation, intraoperative finding, intraoperative time, and anastomotic leak had no statistically significant association with mortality. But patients with intraoperative shock had higher chance of death than patients without intraoperative shock (*p*=0.020) Table 7.

The mean duration of follow up of our patient was 14 months ranging from 3 months to 29 months. All patients were seen at least once. Within 12 months of follow up, no recurrent attack was documented among patients with unresected sigmoid colon. All the patients with colostomy were readmitted for colostomy reversal. Patients stayed on an average of 20 weeks before colostomy closure (ranging from 12 to 30 weeks).

## 4. Discussion

Studies showed ISK being common in the region called ‘‘volvulus belt,” which includes Africa, South America, Russia, Eastern Europe, the Middle East, India, and Brazil. It is also common in Turkey [1, 3, 4, 8, 9, 18, 19]. The condition is seen in relatively young adults, and mean age ranges from 35.8 to 55 years, which is also the case in our study [1, 3, 4, 6–11, 13, 15]. Similar to primary small bowel volvulus and sigmoid volvulus, for reasons not yet clear, ISK is predominantly a male disease. Our study, other studies from Ethiopia, and similar studies elsewhere showed a male to female ratio ranging 3 : 1 to 5 : 1 [1, 3, 6, 10–12, 15, 18].

In developing nations there is a significant delay in patients' presentation to hospitals, even in acute abdomens [3, 11]. It is reported that patients with ISK present relatively earlier but delays of up to 4 days is not uncommon. Literatures showed a mean duration of illness at presentation ranging 0.9–4.4 days [1–4, 6–11, 15]. Our patients mean (1.6 days) was in that range. This study also demonstrated that late presentation was by far common in those who reside in rural area than in urban. This reflects lack of emergency surgical care in most of the rural Ethiopia. The major presenting features of ISK in our series were abdominal pain/tenderness, vomiting, failure to pass feces and/or flatus, abdominal distention, and hypoactive bowel, which are not different from findings of similar studies [1–4, 6–11, 15, 18, 19]. Shock is the presenting feature in up to 60% of the ISK patients [2, 4, 8, 12, 15]. In contrast to this, our series and a Kenyan study by Ooko et al. found relatively less rate of shock at presentation, 17.9% and 16.4%, respectively [3].

Because there is no specific clinical or radiological means to make the diagnosis of ISK, correct preoperative diagnosis is difficult in most cases. The reported accuracy of correct preoperative diagnosis ranges from 0% to 71% [3, 7, 9, 11, 12, 15]. Features of double bowel obstruction and dilated large and small bowel loops with multiple air fluid level, in a patient with clinical signs of bowel of obstruction should be an important indicator of the possible diagnosis of ISK. A high index of suspicion in regions, where volvulus is common, is another important consideration to make an accurate preoperative diagnosis. In our case, nearly one-fifth of the patients were diagnosed preoperatively, which might be related to all the factors described above. Due to patient's presentation with all the cardinal signs of bowel obstruction, which are not specific, small bowel obstruction was the leading misdiagnosis in our series and other studies [1, 6, 7, 9].

The double closed loop nature of the obstruction in ISK leads to early bowel strangulation. The rate of strangulation reported in literature ranges from 73.5 to 93.9% and commonly involves both segments (the ileum and sigmoid) [1–9, 14, 17]. The same is also seen in our patients. Our study and a study performed by Ooko et al. revealed that the rate of strangulation in patients presenting within 24 hours and later to be the same [3]. Raveenthiran also revealed no correlation between the duration of symptoms and the rate of gangrene [15]. In agreement with other reports most of our patients (92.8%) found to have the ileum as the active component of the knot [2, 4, 8, 9, 13].

Even if the type of procedure depends on intraoperative findings and patients' condition, resection of the gangrenous segment with primary anastomosis of the ileum and sigmoid end colostomy is commonly performed in this series and other studies [1, 4, 6, 7, 11–13, 17]. Some authors reported that primary anastomosis of the sigmoid is a safer option provided that the patient is stable and a tension free anastomosis is possible [1–3, 5, 7, 15]. If stoma is indicated, Atamanalp SS recommends to perform colostomy rather than ileostomy or double segment stoma as these are associated poor outcomes [1]. Due to the risk of spillage of toxic bowel content and risk of significant increase in the duration of procedure, the knot is resected en-bloc in our patients with double segment gangrene [1–5, 9, 11, 12].

In patients with gangrenous ileum but viable sigmoid, resection of the ileum should be performed. The sigmoid can be decompressed intraoperatively for facilitation of abdominal closure. Choice of reconstruction of the bowel depends upon the length of the distal ileum when there is enough length to do ileoileal anastomosis that is very anatomic and a preferred choice. Fortunately, all our patients underwent ileoileal anastomosis. The other options of bowel continuity are ileotransverse anastomosis and ileoascending anastomosis [2, 4, 6–9, 13, 15].

It is very uncommon for patients who have ISK not to have strangulation of one or both segments of the knot. It is not clear why few patients remain to have viable bowel despite most having gangrenous changes of both segments. Time of presentation can be an important factor; but in our study, one of the patients presented within 12 hours, while the other presented 48 hours after the onset of the illness [2, 9]. When the bowels are viable, the need for resection is not there. To avoid complication that may arise from resection of bowel, if it is technically possible, untying should be suffice and that is what is performed in our patients. In areas where sigmoid volvulus is common and the sigmoid is redundant, sigmoid resection and primary anastomosis can be performed after untying the knot if patients condition allows and the surgeon is experienced [3, 4, 7, 9–11, 15, 19]. A prophylactic sigmoidopexy or mesosigmoplasty should be avoided as they are not effective [18, 20].

The rate of complications in our study is higher than studies performed by Ooko et al. (24.6%) and Atamanalp SS (20.0–21.6%) [1, 3, 7]. In contrast, the rate is lower than another Ethiopian study performed by Kotisso and Bekele which is 60% [11]. Surgical site infection is reported as the major postoperative complication in most studies and our finding showed the same [3, 4, 6–9]. In our study, anastomotic leak entirely occurred in patients with double segment gangrene. Authors reported higher rate of complications in this group [1, 2, 4, 7, 9]. Our study also found that anastomotic leak is higher in patients who had intraoperative shock. This advocates employment of stomas rather than primary anastomosis in unstable patients [1–3, 6, 8, 9, 14, 17–19]. Furthermore, damage control laparotomy should be initiated for patients with sustained hemodynamic instability despite adequate resuscitative measures. Resection of the gangrenous segment and closure of the bowel edges with suture or staples followed by lavage and temporary abdominal closure are the critical steps. Based on patient's response to resuscitation, planned second look relaparotomy should be performed within 24–48 hours [3, 21].

Overall mortality in ISK can be as high as 66.6% [1–4, 7–13, 15]. Factors such as advanced age, presence of gangrenous bowel, late presentations and diagnosis, comorbidities, pregnancy, and shock (at presentation or after the induction of anesthesia) are associated with increased mortality [1–15]. Our study reported a relatively lower rate of death than most of the studies. This might be due to the younger age of the subjects and the absence of associated comorbid illnesses in our patients. Ooko et al. and Atamanalp SS advocate better perioperative (critical) care as one factor to the reduce mortality. [2, 3]. This study and others studies revealed multiple organ failure (MOF) due to septic shock as the major cause of death [1–4, 7–13, 15]. In our study, intraoperative shock was the only factor significantly associated with increased mortality (*p*=0.020). The reported mean hospitalization ranges between 7 and 14 days, which is in line with our finding (10.7 days) [3, 6–8, 15].

In summary, ISK is uncommon cause of acute intestinal obstruction with nonspecific presentation of bowel obstruction. Though accurate preoperative diagnosis may not change the decision to do emergency laparotomy, high index suspicion is required for preoperative diagnosis of the disease. ISK is associated with early onset bowel strangulation, which results in significant morbidity and mortality. Vigorous fluid resuscitation followed by urgent and effective laparotomy are the pillars of treatment. The choice of surgical procedure is determined by intraoperative bowel status and patients' general condition. Intraoperative hemodynamic instability (shock, SBP < 90 mmHg) is associated with increased mortality.

We recommend that ISK should be considered as a differential diagnosis in patients presenting with acute intestinal obstruction, especially in areas where sigmoid volvulus is common. Moreover, resection followed by stomas should be employed in unstable patients. Patients with intraoperative instability deserve aggressive resuscitation and possible damage control surgery with aggressive postoperative care in the ICU.

## Figures and Tables

**Table 1:** Sociodemographic characteristics of patients operated for ISK at SPHMMC, Addis Ababa, Ethiopia, from February 2014 to January 2020.

Variables		Frequency (*n* = 28)	Percentage
Age	<20	1	3.6
	20–29	9	32.1
	30–39	5	17.8
	40–49	4	14.3
	50–59	3	10.7
	60–69	2	7.2
	≥70	4	14.3

Sex	Male	21	75
	Female	7	25

Residence	Urban	8	28.6
	Rural	20	71.4

**Table 2:** Clinical Presentation of patients operated for ISK at SPHMMC, Addis Ababa, Ethiopia, from February 2014 to January 2020.

Clinical presentation	Frequency	Percentage
*Symptoms*		
Abdominal pain	28	100
Vomiting	28	100
Abdominal distention	24	85.7
Failure to pass flatus or feces	23	82.1

*Signs*		
Shock at presentation	5	17.9
Abdominal tenderness	26	92.8
Guarding/Rebound tenderness	19	67.8
Hypoactive bowel sound	15	53.6
Normoactive bowel sound	7	25
Hyperactive bowel sound	6	21.4
Hypertympanic abdomen	24	85.7
Stool in the rectum	15	53.8
Empty rectum	11	39.3
Blood in the rectum	2	7.1

**Table 3:** Preoperative diagnosis of patients operated for ISK at SPHMMC, Addis Ababa, Ethiopia, from February 2014 to January 2020.

Preoperative diagnosis	Frequency (*n* = 28)	Percentage
Small bowel obstruction	14	50.0
ISK	6	21.4
Sigmoid volvulus	4	14.2
Perforated viscus	2	7.2
Acute mesenteric ischemia	1	3.6
Acute pancreatitis	1	3.6

**Table 4:** Intraoperative finding and type of procedure performed for patients operated for ISK at SPHMMC, Addis Ababa, Ethiopia, from February 2014 to January 2020.

Finding at laparotomy	Procedure performed	Frequency (*n* = 28)	Percentage
Gangrenous ileum and gangrenous sigmoid colon	Ileal resection and primary ileoileal anastomosis, sigmoid resection and Hartman's procedure	16	57.1
	Ileal resection and ileostomy, sigmoid resection, and Hartman's procedure	1	3.6

Gangrenous ileum and viable sigmoid colon	Resection of the ileum with primary ileoileal anastomosis and rectal tube sigmoid decompression	8	28.5

Viable ileum and gangrenous sigmoid colon	Hartman's procedure	1	3.6

Viable ilium and viable sigmoid	Untying	2	7.2

**Table 5:** Outcome of patients operated for ISK SPHMMC, Addis Ababa, Ethiopia, from February 2014 to January 2020.

Outcome	Frequency	Percentage
*Complications*		
ARDS	5	17.8
Deep surgical site infection	4	14.3
Superficial surgical site infection	3	10.7
Complete wound dehiscence	3	10.7
Anastomotic leak	3	10.7
Acute renal failure	2	7.2
Hospital acquired pneumonia	1	3.6

*Condition on discharge*		
Improved	22	78.6
Died	6	21.4

**Table 6:** Outcome of operated patients for ISK in relation to intraoperative findings SPHMMC, Addis Ababa, Ethiopia, from February 2014 to January 2020.

Finding at laparotomy	Improved		Died		Total	
	*n*	%	*n*	%	*n*	%
Gangrenous ileum and sigmoid colon	13	76.4	4	23.5	17	60.7
Gangrenous ileum only	7	87.5	1	12.5	8	28.5
Gangrenous sigmoid colon only	0	0	1	100	1	3.6
Viable ileum and sigmoid colon	2	100	0	0	2	7.2

**Table 7:** Factors associated with mortality in patients operated for ISK, SPHMMC, Addis Ababa, Ethiopia, from February 2014 to January 2020.

Variables	Outcome (died)		*p* value
	Yes	No	
Age			0.072
<60	3	19	
>=60	3	3	

Sex			0.599
M	5	16	
F	1	6	

Residence			0.475
Urban	1	7	
Rural	5	15	

Duration of presentation			0.171
<24 hrs	1	11	
>=24 hrs	5	11	

Gangrenous bowel			0.940
Double segment	4	13	
Single segment	2	7	

Intraoperative duration			0.171
<3 hrs	1	12	
>=3 hrs	5	10	

Intraoperative SBP			0.020^*∗*^
<90 mmHg	3	1	
>90 mmHg	3	21	

Anastomotic leak			0.107
Yes	2	1	
No	4	21	

^*∗*^Significantly associated at *p* value <0.05.

## Data Availability

The data used to support the findings of this study are available from the corresponding author upon request.
